# Non-Invasive Preimplantation Genetic Testing

**DOI:** 10.3390/genes16050552

**Published:** 2025-04-30

**Authors:** Daniela N. Bakalova, Luis Navarro-Sánchez, Carmen Rubio

**Affiliations:** Igenomix (Part of Vitrolife Group), R&D Genetic Services, 46980 Paterna, Valencia, Spain; dany.bakalova@igenomix.com (D.N.B.); luis.navarro@igenomix.com (L.N.-S.)

**Keywords:** non-invasive PGT, cell-free DNA, aneuploidy, blastocyst, culture medium

## Abstract

To minimise the influence of chromosomal abnormalities during IVF treatment, embryos can be screened before transfer using preimplantation genetic testing. This typically involves an invasive trophectoderm biopsy at the blastocyst stage, where 4–8 cells are collected and analysed. However, emerging evidence indicates that, as embryos develop in vitro in culture media, they release cell-free DNA into the media, providing an alternative source of genetic material that can be accessed non-invasively. Spent blastocyst media samples that contain embryo cell-free DNA demonstrate high informativity rates and ploidy concordance when compared with the corresponding trophectoderm, inner cell mass, or whole blastocyst results. However, optimising this non-invasive approach requires several changes to embryo culture protocols, including additional embryo washes to tackle contamination and extending embryo culture time to maximise the amount of cell-free DNA released into the culture media. In this review, we discuss this novel non-invasive approach for aneuploidy detection and embryo prioritisation, as well as the current data and future prospects for utilising cell-free DNA analysis to identify structural rearrangements and single gene disorders.

## 1. The Fundamentals of Embryo Chromosomal Abnormalities

Aneuploidy refers to a phenomenon where the total number of chromosomes in a cell deviates from the wild-type complement (23 pairs; 46 chromosomes). Broadly, this can manifest as an additional chromosome or segment of a chromosome (trisomy or partial gain), a missing chromosome or segment of a chromosome (monosomy or partial loss) or combinations of both [1]. Cells rely upon specific mechanisms, such as spindle assembly checkpoints, cell cycle regulation, and cohesion complexes—all of which are responsible for accurate chromosome segregation during mitosis and meiosis—to ensure they retain a euploid state [2,3]. When errors occur during these processes, aneuploidy can develop.

The majority of aneuploidies in embryos can be traced to a maternal origin, with more than 90% of errors originating during oogenesis due to maternal meiosis I impairments [1,4]. Indeed, the incidence of embryo aneuploidy rises exponentially for women over 35 years of age, reaching as high as 70% in patients over the age of 43. Interestingly, age does not seem to play such a significant role in male reproductive potential, with only 1–8% of spermatozoa reported to be aneuploid in an age-independent manner [5,6]. Although there is evidence to suggest that advanced paternal age (APA) contributes to an increased risk of miscarriage and time to pregnancy in natural conceptions, the impact of APA in assisted reproductive cycles is poorly defined and remains contradictory [7]. Discrepant findings are also reported in terms of sperm DNA damage. Nevertheless, male factor infertility is a recognised indication for fertility treatment [8].

Aneuploidy has been associated with poor embryo implantation, increased rate of miscarriage and higher risk of foetuses born with chromosomal abnormalities [9,10,11,12]. Due to the genetic disruption from the loss or gain of chromosomal content, many aneuploidies—such as monosomies—are generally incompatible with life. However, trisomies of certain chromosomes can lead to live births of children affected by a chromosome disorder (e.g., Trisomy 13: Patau syndrome, Trisomy 18: Edwards syndrome, Trisomy 21: Down syndrome, and sex aneuploidies).

In addition to numerical chromosomal abnormalities, embryos may also be affected by aberrant chromosomal structural rearrangements (SR) that arise from recombination, repair, or replication errors. There are four types of SR: deletions and duplications (del/dup), inversions, and translocations. Del/dup may appear de novo in the embryo, or by inheritance in cases where one or both parents are carriers of a balanced translocation (reciprocal/Robertsonian) or inversion. Most frequently, carriers of balanced structural rearrangements are phenotypically normal; however, their gametes may be unbalanced, leading to unbalanced/affected embryos that, if transferred, may result in miscarriage or live-born offspring with physical or cognitive impairments [13,14].

The primary goal of reproductive treatment is to achieve a healthy outcome for the mother and newborn. Given the association between chromosomal numerical/structural abnormalities and implantation failure/miscarriage, it is important that embryos transferred in an assisted reproductive technology (ART) cycle have the correct number of chromosomes.

## 2. Identifying Chromosomal Abnormalities in Preimplantation Embryos: The Old, the New, and the Future

During assisted fertility treatment, embryologists perform an assessment of blastocyst morphology as a means of identifying well-developing embryos, which can then be prioritised for transfer. However, some embryos affected by chromosomal aneuploidies, structural rearrangements, or genetic disorders may still have good morphological scores, meaning these embryos may be selected to undergo transfer to the uterus. In other words, conventional morphological analysis does not prevent the transfer of abnormal embryos [15,16]. Thus, additional screening measures are necessary to avoid the transfer of non-euploid embryos in ART.

A different approach to traditional morphology-based assessment is morphokinetics. Unfortunately, early-development morphokinetic parameters do not appear to have the capacity to accurately predict abnormalities such as aneuploidy [17], though morphological events during the latter stages of embryo development show more potential in identifying embryo ploidy status [18,19,20,21]. Several studies have failed to find a general relationship between morphokinetics and aneuploidy [22,23], while others reported differences in compaction rates, time to start blastulation, time to reach blastocyst expansion, and time to blastocyst hatching between euploid and aneuploid embryos [18,19,20,21]. However, embryo selection according to these factors did not improve clinical outcomes [20,21]. Morphokinetic parameters have not been investigated as a tool to identify and select embryos affected by structural rearrangements or monogenic disorders.

Preimplantation genetic testing for aneuploidies (PGT-A) provides a comprehensive, robust, and reliable alternative to these methods, accurately identifying aneuploidies in preimplantation embryos. Indeed, there are several potential advantages to using PGT-A alongside ART: increased ongoing pregnancy rate, reduced time to conception and transfers to achieve a live birth, and lower miscarriage rates [24,25,26,27]. Moreover, research suggests that average costs of fertility treatment for some patient groups can be lowered by judicious use of PGT-A [27,28]. These advantages are especially relevant in cases with advanced maternal age, where the risk of aneuploidy is high and the availability of oocytes may be lower [28,29].

PGT-A can be applied to different preimplantation developmental stages by collecting a small biopsy sample from either the first and second polar bodies (PB), the blastomeres from cleavage-stage embryos, or the trophectoderm (TE) at the blastocyst stage. Although there is some variation in practice across different geographical and clinical settings, the most common approach for PGT-A is TE biopsy followed by analysis via next-generation sequencing (NGS) [12,30,31,32]. Using this method of embryo biopsy, it is also possible to test for structural rearrangements (PGT-SR) and monogenic disorders (PGT-M). Interestingly, some centres are adopting a more dynamic embryo prioritisation system that combines the PGT result and morphology grading in order to establish which blastocyst has the highest implantation potential [33].

In recent years, there has been a slow and cautious movement towards investigating the possibility of performing embryo aneuploidy assessment without the need for TE biopsy. Blastocentesis has been proposed as one such approach; this requires aspiration of the blastocoel fluid (BF) [34] at the expanded blastocyst stage, without removing any embryonic cells. Although this appears to be a promising technique, it is speculated that the DNA in BF originates, at least in part, from cells that are necrotic or apoptotic, which could compromise DNA quantity or integrity [35,36]. However, it should be noted that the mechanisms and true origin of BF-derived DNA are not fully understood, and as such, it is possible that not all DNA is derived from apoptotic pathways. There is significant variation in reported amplification rates and concordance of BF-based PGT-A with the corresponding TE biopsy (56–82% and 37–97%, respectively) [37,38,39,40]. This variation likely stems from differences in technical procedures and level of experience between the technicians performing blastocoel collapse and BF collection [39]. Thus, despite the potential clinical value [41], the application of blastocentesis is not widespread.

Blastocentesis and conventional PGT require embryo manipulation; in both cases, the zona pellucida must be breached by laser or acid to retrieve cells or fluids from the embryo. Technicians who perform sample collection must undergo specialised training, which, coupled with high equipment costs (e.g., laser), may reduce wider implementation and accessibility of PGT. Additionally, the possibility of harming the embryo is a concern raised by clinicians and patients alike, with recently published studies linking TE biopsy with a significant increase in preeclampsia [42], pre-term birth [43], and hypertensive disorders amongst pregnant women [44]. A scoping review investigating the potential clinical, neonatal, and long-term impacts of embryo biopsy techniques in a PGT setting reported conflicting evidence [45]. While there are some data describing negative outcomes associated with TE biopsy, it should be noted that this risk is minimal in the hands of experienced technicians, as demonstrated by several groups who have deemed the biopsy strategy safe in relation to implantation potential, live birth rate, and neonatal outcomes [11,29,46,47,48].

**Figure 1:**
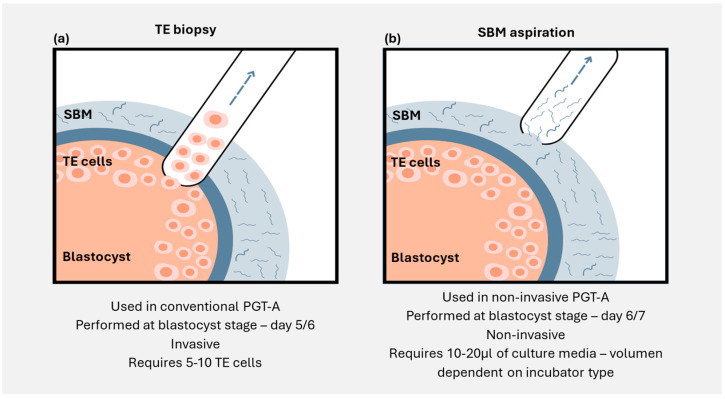
Illustration showing the differences between (**a**) trophectoderm biopsy and (**b**) spent blastocyst media aspiration. TE: trophectoderm; SBM: spent blastocyst media.

The prospect of non-invasive PGT (niPGT) is therefore an exciting opportunity to negate the requirement of invasively sourcing genetic material directly from the embryo (Figure 1). Ideally, such an approach would offer the possible benefits of conventional PGT—improved live birth rate, reduced rate of miscarriage and multiple pregnancies, and shorter time to pregnancy—while avoiding the potential risk and cost associated with embryo manipulation and biopsy. In this chapter, we review the use of cell-free DNA (cfDNA) found in the culture media in which an embryo has grown—the spent blastocyst media (SBM)—as an alternative, non-invasive source of genetic material for the analysis of chromosomal abnormalities in preimplantation embryos. We evaluate the available concordance studies between SBM and the corresponding blastocyst, the different embryo culture protocols, methodologies for analysis and interpretation, and the origin of cfDNA and its potential clinical application as an objective biomarker to support embryo transfer prioritisation.

## 3. Embryonic Cell-Free DNA Analysis: How Close Are We to an niPGT Solution?

Cell-free DNA is found in the culture media in which embryos grow and develop in the IVF laboratory. Stigliani et al. were the first group to work with embryonic cfDNA, correlating a higher content of cfDNA in the culture media with an increased fragmentation rate on days 2 and 3 of embryo development—an important morphological parameter for embryo implantation [49]. The same group later reported that a high ratio of cfDNA in culture media from day 3 embryos was associated with higher blastulation and embryo implantation outcomes, especially when combined with morphological grading [50].

A small number of monogenic disorders have been investigated using a cfDNA approach—niPGT-M. Notably, Calluzzi et al. were the first to genotype the C677T polymorphism of the *MTHFR* gene, which is associated with various diseases, including cardiovascular illness, cancer, and diabetes. The group obtained informative results in 62.5% of samples (5/8), showing promising data for further research [51]. Monogenic disorders such as α-thalassemia and β-thalassemia have also been studied using cfDNA analysis, achieving high SBM amplification rates [52,53]. However, niPGT-M is hindered by the risk of maternal cell contamination, with some studies reporting the presence of the affected parental genotype in the SBM, while the TE biopsy was determined to be unaffected [54]. Given the complexity of single-gene disorders, as well as the significant implications in cases of misdiagnosis, further research and optimisation are required before this test can be applied in a clinical setting.

There are very limited data—in both the number of studies and sample size—regarding non-invasive analysis for structural rearrangements (niPGT-SR). One group who performed niPGT-SR in a couple who carried a balanced translocation were able to successfully detect the relevant del/dup in an SBM sample [55]. Although this was a promising proof of concept, the sizes of the del/dup were large (12 Mb and 45 Mb). More samples, with smaller anomalies, are needed to determine the reliability of this approach and detection thresholds.

The majority of research relating to non-invasive embryo assessment via cell-free DNA analysis focuses on aneuploidy screening, otherwise known as non-invasive PGT-A (niPGT-A).

### 3.1. Cell-Free DNA Analysis for niPGT-A: Concordance Studies

The concept of adopting cfDNA for niPGT-A was first recognised by Shamonki et al. in 2016. The group demonstrated promising concordance between SBM and TE biopsies taken from the corresponding embryos, hence providing a proof of concept that cfDNA could be used to detect embryonic aneuploidy [56].

**Figure 2:**
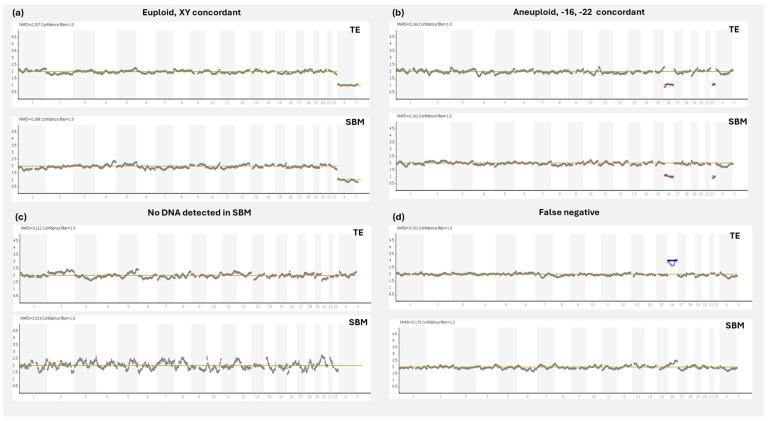
NGS concordance profiles between corresponding SBM and TE samples. (**a**) Concordant trace where both SBM and TE samples present with a euploid male profile. (**b**) Concordant trace where both SBM and TE samples present with aneuploid profiles, with losses in chromosomes 16 and 22. (**c**) Euploid result in TE, but no DNA detected result in SBM, as demonstrated by missing sex chromosomes. (**d**) Discordant trace displaying a false negative result, where the TE is showing a gain in chromosome 16, but the aneuploidy is not detected in the SBM. TE: trophectoderm; SBM: spent blastocyst media; NGS: next generation sequencing.

Since then, several groups have investigated the possible application of cfDNA for clinical niPGT-A by comparing SBM results with those from a corresponding embryo sample. The primary parameters measured in these studies were the informativity rate of SBM (percentage of diagnosable SBM samples), the ploidy concordance rate (matching euploid/aneuploid results between the SBM and reference sample), the false positive rate (SBM is aneuploid and the reference sample is euploid), and the false negative rate (SBM is euploid and the reference sample is aneuploid) (Figure 2).

The most common reference sample used in concordance studies is TE biopsy. This is primarily due to the practicality of collecting and sending the SBM for concordance analysis at the same time as sending the TE biopsy for clinical PGT-A testing. SBM informativity and SBM-TE ploidy concordance rates show high variability, ranging between 62.7–98.2% and 30.8–89.6%, respectively [57,58,59,60,61,62,63,64,65,66,67,68,69]. Only one study has compared SBM to polar body biopsy, obtaining an 81.8% SBM informativity rate and a 72.2% SBM-PB ploidy concordance rate [70].

Some groups used the whole blastocyst (WB) as the gold standard for comparison. Xu et al. [55] used 42 vitrified day 3 embryos that were thawed and cultured until day 5; all SBM samples provided informative results, with SBM-WB concordance of 85.7%. Ho et al., Huang et al., and Shitara et al. [58,59,64] compared SBM with WB, obtaining high informativity rates for the SBM (97.6%, 92.3%, and 95%, respectively) but disparate concordance rates: 45.5%, 93.7%, and 93.8%, respectively. A more recent study investigated the impact of blastocyst quality on SBM informativity rate and ploidy concordance—though all embryos provided informative SBM, those with high quality displayed higher SBM-WB concordance compared with low quality (70.8% vs. 59.1%, respectively) [71].

An interesting new approach to niPGT-A utilises previously vitrified blastocysts. Frozen day 5/6 blastocysts are thawed according to standard practice, washed in fresh media, and cultured for an additional 8–24 h (8 h for day 6 blastocysts; 24 h for day 5 blastocysts) before the SBM is aspirated and analysed for chromosomal aneuploidy. This method can be beneficial in several ways. Firstly, developing a protocol for previously vitrified blastocysts allows greater patient accessibility for aneuploidy screening that may otherwise not be possible. Secondly, vitrified-thawed niPGT-A can rescue embryos that have previously been tested using conventional PGT-A where the TE biopsy did not yield a result. In many cases, clinics are hesitant to perform re-biopsy for additional PGT-A analysis, and as such, the non-informative embryo may be de-prioritised for transfer despite the possibility of being euploid—or transferred with the risk of being aneuploid. Yin et al. worked with 75 thawed and re-cultured (for 24 h) day 5/6 embryos, where TE biopsy had already been performed. They achieved SBM informativity of 78.7% and SBM-TE concordance of 89.8% [72]. Other groups have also investigated this protocol, achieving SBM informativity in the range 74.3–81.5% and ploidy concordance between 61.9 and 92.5% [73,74,75].

Despite the different embryology protocols adopted by research groups, three principles remain consistent: (i) careful oocyte denudation is performed to remove maternal cumulus cells; (ii) embryos are washed and moved to an individual culture drop for the final stages of development before media collection; and (iii) embryos are cultured for an extended period of time. Variations in these steps introduce opportunities for dissimilarities in concordance and contamination rates.

Due to the required protocol modifications, IVF centres must consider undergoing a validation process with the relevant testing provider to ensure that contamination is not present and SBM-reference sample concordance rates are high prior to the implementation of the niPGT-A approach. Validations are also a relevant aspect, as they allow clinics to confirm that the required protocol modifications are not impacting embryo quality, viability, and developmental potential; research demonstrates that these modifications have no deleterious impact, as will be discussed later in this review. While some research groups have considered whether additional embryo manipulation might improve niPGT results, neither performing assisted hatching nor working with previously biopsied embryos has shown any benefit [58,75].

### 3.2. Minimally Invasive Embryo Analysis

Several studies have also assessed the combination of SBM and blastocoel fluid; this is a method referred to as minimally invasive PGT (miPGT). Unlike blastocentesis, where BF is aspirated from the blastocoel, in miPGT, the blastocoel is collapsed, releasing its contents into the SBM. This means that the miPGT sample analysed is a combination of SBM and BF. In addition to this mandatory blastocoel collapse, various studies have conducted extra embryo manipulations such as TE biopsy, vitrification, and/or assisted hatching as part of their protocols. In these cases, for the same embryo, NGS results from BF plus SBM were compared to PGT-A results from TE biopsy and WB. The number of samples analysed in all cases was low, and the results were heterogeneous: the informativity rate of SBM + BF ranged between 87.5 and 100%, with variable concordance rates between the sample pairs (SBM + BF and TE/WB), ranging between 76.3–98.3% and 70.4–100%, respectively. Though minimally invasive, this approach did not provide an advantage: no benefit was observed when collapsing the blastocoel to retrieve BF, as the use of BF in combination with SBM did not provide better results than analysing the SBM on its own [76,77,78,79,80,81,82].

Nevertheless, this minimally invasive approach was used by one group to study embryos created by couples who were known carriers of structural rearrangements. A total of 41 embryos collected from 22 couples resulted in a 100% SBM + BF informativity rate and a 90% SBM + BF concordance rate with the corresponding TE biopsy. The sizes of the del/dups ranged between 4.1 and 76 Mb, suggesting that miPGT-SR had the potential to detect small size abnormalities [78]. Due to the limited sample size, additional research is required to confirm these findings.

## 4. Origin of Embryonic Cell-Free DNA in Spent Blastocyst Media

The true origin and mechanisms of embryonic cfDNA release into spent blastocyst media are still not entirely understood. The first hurdle is to show that the presence of cfDNA in culture media is determined by exposure to an embryo—this has been proven by negative control studies, which demonstrate higher levels of cfDNA in media samples that have hosted embryos than in those that have not [57]. Some groups speculate that the presence of DNA in the base culture medium could be due to protein supplementation components such as human serum albumin (HSA) [83]; however, it appears that this DNA does not impact downstream processes and the chromosomal screening result. It should be noted that the quantity of cfDNA in SBM samples is significantly higher than in control media samples, with an increasing amount of cfDNA reported in SBM samples that have been exposed to an embryo for a longer period of time. In 2018, Vera-Rodriquez et al. performed quantitative PCR (qPCR) on SBM samples, reporting an average amount of cfDNA in 20 µL of culture media of 6.7 pg, versus only 1.4 pg in the control samples [57]. A different group utilised four different methodologies—digital PCR (dPCR), long-range PCR, qPCR, and DNA fingerprinting—to determine the amount of cfDNA in SBM from 227 blastocysts. Higher levels of DNA were observed in SBM samples exposed to an embryo compared to media controls, with the levels increasing at the blastocyst stage [83]. These studies suggest that the majority of cfDNA in SBM originates from embryos and accumulates as embryos grow and develop in the medium drop.

There is insufficient evidence regarding the mechanisms behind cfDNA release into the SBM, with many questions still unanswered, but several groups have studied this and proposed a number of plausible theories. Firstly, some literature suggests that cfDNA could be secreted through apoptotic pathways since embryos undergo rapid dynamic changes during late preimplantation development that result in a high loss and gain of cells [84]. This hypothesis would be consistent with the origin of cfDNA from apoptotic placental cells used in non-invasive prenatal testing; however, it is unclear if the same principle can be applied to cfDNA found in SBM. Kuznyetsov et al. obtained similar cfDNA quantities and sizes of amplified DNA fragments from embryos with different morphological grades, suggesting that apoptosis and necrosis were not likely to be the only mechanisms driving cfDNA release [80]. Others propose that the release of cfDNA is a more active process, in the form of extracellular vesicles which pass through the zona pellucida (ZP) [85,86]. Moreover, recent studies focusing on the nuclear dynamics of human blastocysts have observed new phenomena (“nuclear buds”) during embryo development, which may imply a constitutive mechanism that occurs during embryonic evolution and could explain cfDNA release [87].

While the role of polar bodies in contributing to SBM cfDNA has been discussed [57], research supports the idea that the cfDNA is primarily sourced from the TE and inner cell mass (ICM) of the developing embryo. A recent prospective study investigated this hypothesis, with the results demonstrating 86.1% and 89.6% ploidy concordance between SBM-ICM and SBM-TE biopsies, respectively [88]. One study assessing genome-wide DNA methylation and sequencing in SBM samples from day 6 blastocysts without cumulus cell or polar body contamination revealed that one-third (18/61) of the cfDNA had methylation patterns that aligned with the TE and two-thirds (43/61) with the ICM, suggesting that embryonic cfDNA can be derived from both parts of the embryo (*p* < 0.01) [66]. In another study, frozen-thawed embryos were used to analyse whole chromosome copy number, with comparable concordance rates observed between SBM and TE biopsy, SBM and WB, and TE biopsy and WB [76]. Collectively, these data show very similar, high concordance rates of cfDNA with all three reference samples, thus indicating that the true chromosomal content of the embryo is well represented in the SBM.

## 5. Methodology: Protocol Modifications

**Figure 3:**
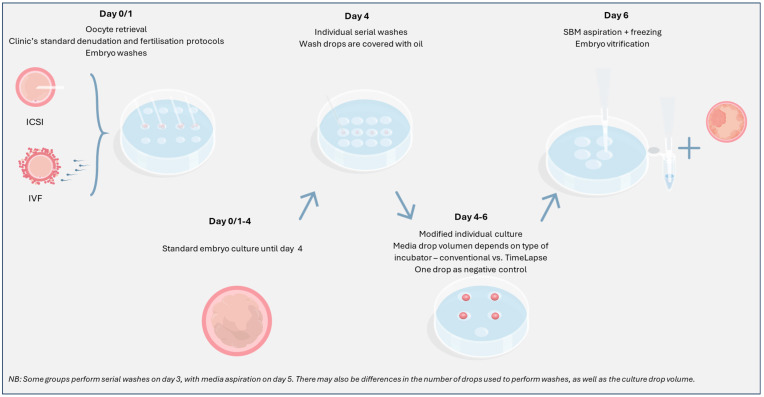
Brief overview of a general non-invasive PGT embryo culture protocol. SBM: spent blastocyst media; ICSI: intracytoplasmic sperm injection; IVF: in vitro fertilisation.

To achieve optimal results following analysis of cell-free DNA from spent blastocyst media, personnel in the IVF laboratory should consider the following key protocol modifications (Figure 3):

### 5.1. Managing Contamination

It is essential to minimise DNA contamination of SBM in order to ensure that only embryonic cfDNA is amplified and analysed. Contamination may be maternal—arising from residual cumulus cells following oocyte denudation (i.e., maternal cell contamination; MCC) or exogenous—arising from external sources such as technicians or contaminated materials/working surfaces [61,62,89]. Avoiding contamination is one of the greatest challenges in niPGT [55,57,58,60,61,62,66]; therefore, careful embryo manipulation and protocol adherence are critical to reduce the risk of maternal/external DNA impacting the niPGT result, as either type of contamination could lead to a false negative result.

Groups that have researched or clinically applied niPGT have proposed several steps to minimise the risk of contamination. These include thorough oocyte denudation (prior to ICSI/following IVF) to reduce maternal cell contamination, serial embryo washes to remove any residual cumulus cells, culture media changes to reduce the presence of unwanted degraded DNA, working in a clean area using appropriate UV decontamination and personal protective equipment to reduce external contamination, and culturing embryos individually to avoid cross-contamination [61,62].

The opportunity for external contamination from plasticware and culture media is difficult to quantify. One group reported consistent low-level DNA contamination in media controls which had not been exposed to an embryo. The authors proposed that this was introduced into the media during the manufacturing process or as a result of high DNA-binding-affinity protein supplementation, perhaps suggesting that the specific type of media used for niPGT should be carefully selected [83]. However, the data are conflicting; a large multi-centre study demonstrated no differences in SBM-TE concordance rates between different media brands/types [62].

For additional security, it is recommended that technicians include a negative control for each patient that undergoes niPGT. This consists of one drop of media that has been cultured alongside the embryos, following the same protocol, without coming into contact with an embryo. If there is no DNA present in the negative control following analysis alongside SBM samples, then the clinic can be confident that the niPGT results are unlikely to have been affected by contamination.

Beyond these intuitive, straightforward recommendations to minimise contamination, different research protocols/testing providers have notable variations in their key steps. For example, some laboratories offering niPGT-A recommend that an additional embryo wash is performed on day 3, following standard embryo culture until this point, whereas others suggest washing on day 4. There are also differences between the number of drops embryos should be washed in, the drop volume, and whether this should be carried out in group washing conditions or individually [90]. Moreover, there is variation in protocol compliance between IVF centres that use niPGT, with some choosing to omit the denudation step or additional washes, which may impact downstream processes. Performing thorough washes on day 4, followed by media aspiration on day 6, all while working in a clean environment, is suggested to be one of the optimal protocols to minimise the risk of contamination.

### 5.2. Extended Embryo Culture

Since the initial discovery that cell-free DNA from SBM can be used to establish an embryo’s aneuploidy status, the optimal time for media collection has been highly debated. Some groups have established that collection on day 6 provides the most reliable results, whereas others continue to experiment with how to best optimise the protocol for media aspiration on day 5. Extending embryo culture until day 6 can make a significant difference in SBM informativity and concordance rates with TE, ICM, and WB [59,61,62]. Indeed, SBM collected from embryos that were cultured until day 5, with media change taking place on day 3, showed lower informativity and increased presence of degraded DNA (from residual cumulus cells) that can interfere with the results [57] as compared to SBM collected from embryos cultured until day 6 (day 5 vs. day 6 informativity: Yeung et al.: 55.6% vs. 84.6%; Rubio et al.: 81.8% vs. 98.8%, respectively) [60,61].

In a pilot study including 115 SMB samples and the corresponding TE biopsies, Rubio et al. demonstrated a significantly higher concordance for media collected on day 6/7 when compared with day 5 (84% vs. 63%, respectively) when applying an NGS approach [61]. Indeed, the same observations were made in a later study which investigated concordance rates using two different platforms—NICS (Yikon Genomics, China) and VeriSeq (Illumina). Ploidy concordance rates were notably lower for media collected on day 5 vs. media collected on day 6 across both testing methods: 60.9% on day 5 for both techniques versus 92% (NICS) and 86.5% (VeriSeq) for day 6 embryos [63]. This further supports the notion that extended embryo culture improves concordance rates and thus may be more appropriate for non-invasive testing. Interestingly, one group performed niPGT-A without modifying the clinical protocol, meaning that SBM was collected on the day when TE biopsy was planned, which varied between day 5 or day 6, depending on embryo development. They reported that SBM collected on day 6 had a significantly higher probability of producing an interpretable result compared to SBM collected on day 5 (84.6% vs. 55.6%, respectively). However, ploidy concordance rates were similar, at 76% for day 5 samples and 71.2% for day 6 samples [60].

The main concern with extending culture until day 6 is the impact on embryo viability and reproductive potential. Notably, several studies have shown that in conventional PGT-A cases using euploid day 6 embryos, clinical outcomes were similar to those with euploid day 5 embryos [91,92,93,94,95]. Additionally, in a pilot study using niPGT-A and culturing blastocysts until day 6, pregnancy rates were comparable to conventional PGT-A cases with day 5 or day 6 blastocysts [96].

Recently, Sakkas et al. showed that implementation of niPGT-A protocol modifications does not have a negative impact on blastocyst viability and clinical outcomes: decreasing the drop culture volume, including extra washes to avoid maternal contamination, and performing vitrification for all embryos on day 6 (embryo vitrification is required in order to allow time for testing the SBM, as is the case with conventional PGT-A) did not affect embryo developmental potential [91]. Regarding the reduced culture drop volume required as part of the niPGT-A modified conditions, Maggiulli et al. also did not observe a negative effect on embryo viability and developmental potential [97].

### 5.3. Previously Vitrified Blastocysts

Implementing niPGT-A for previously vitrified blastocysts may give rise to concerns regarding embryo viability following double embryo vitrification and thawing—first vitrification-thaw: initial blastocyst freezing and thaw to re-culture the blastocyst and collect the media sample; second vitrification-thaw: embryo freezing following media collection and embryo thaw ahead of transfer. Studies investigating this are limited in number and usually apply different vitrification methodologies and/or clinical scenarios.

Zheng et al. reported inconclusive evidence—the authors identified a higher miscarriage rate in the double vitrification vs. fresh embryo group (33.93% vs. 19.07%, respectively); however, no differences were observed in neonatal outcomes [98]. Moreover, Aluko et al. have reported a lower chance of achieving a clinical pregnancy or live birth following an embryo biopsy and double-vitrification procedure [99], but it should be noted that these results may not be a reliable representation of the clinical outcomes expected in a niPGT-A protocol, where a TE biopsy would not be performed.

Conversely, a recent report from Al Hashimi et al. demonstrates that double vitrification and warming of blastocysts does not affect pregnancy, miscarriage, and live birth rates after single embryo transfer (SET) [100]. From the 84 euploid double vitrification embryos that were warmed for treatment, 100% survived and were transferred [100]. Furthermore, Theodorou et al. retrospectively reviewed clinical outcomes for 694 single euploid embryo transfers, reporting no differences in pregnancy rate, clinical pregnancy rate, and live birth rate between single and double embryo vitrification groups [101]. Recent, large-cohort studies demonstrate that implementing a niPGT-A program for previously vitrified blastocysts does not hinder the likelihood of achieving and maintaining a pregnancy.

## 6. Benefits of Implementing niPGT-A

Non-invasive PGT-A is an alternative approach that could help patients across the globe, particularly in cases where invasive chromosome testing is not favoured or possible. From a regional perspective, niPGT-A may be especially valuable in countries where legal regulations and/or ethical constraints could limit the use of invasive procedures. For instance, embryo biopsy has only recently been legalised in Germany, primarily for the application of PGT-M; this remains restricted and requires specific authorisation [102]. A similar practice for embryo testing is adopted in most Nordic countries [103].

The need for technologies such as niPGT-A via cell-free DNA analysis is further emphasised in regions/clinics with limited resources (e.g., small clinics with limited funding for equipment), offering a safe and objective assay to support IVF treatment. Clinics in low-income/low-resource countries where fertility treatment is readily offered may also benefit from the implementation of niPGT-A.

Many advocates of niPGT-A propose its use as a prioritisation tool for embryo selection rather than as a direct replacement for TE-based PGT-A—i.e., niPGT-A could provide an objective means of selecting which embryo to transfer first. This prioritisation approach may be advantageous; using the SBM result as guidance for the order in which embryos should be transferred may reduce the number of discarded embryos and provide more embryos with the opportunity of being transferred.

While modifying finely tuned embryo culture systems can present a hurdle to niPGT-A implementation, these adjustments do not have a deleterious impact on outcomes (as already discussed), and the modified protocols have been developed to work with different equipment and methods. For instance, the majority of niPGT-A approaches use a reduced culture volume to maximise the amount of cfDNA. For conventional incubators, volumes between 4 µL and 30 µL drops have been tried and tested [54,55,56,58,59,60,61,62,63,70,76,77,78,104], and for time-lapse systems, 20–25 µL drops have been applied [57] without affecting embryo development and reproductive potential [105].

Moreover, single-step and sequential media from a variety of different brands have been tested, providing similar SBM-TE concordance rates [62]. Similarly, no significant differences have been reported in ploidy concordance rates between humidified or non-humidified systems [62].

Finally, clinics have different standard practices for methods of fertilisation during niPGT-A, with most preferentially using intracytoplasmic sperm injection (ICSI) vs. IVF [68,90]. Research demonstrates that ICSI and IVF have similar sensitivity (87.9% vs. 80.9%) and specificity (69.9% vs. 78.6%) rates when applied to niPGT-A. Interestingly, using IVF is not associated with increased spermatozoa contamination of the SBM [62]. Chow et al. reported that DNA of paternal origin was present in SBM following one day of culture; however, by day 6, the SBM did not contain any paternal DNA [90]. We speculate that any paternal DNA that may originate from spermatozoa following conventional IVF fertilisation is removed during the additional washes on day 3/4 of embryo culture. Furthermore, another group compared three whole genome amplification (WGA) methods that may be applied for niPGT-A for the detection of spermatozoa DNA [106]. Sperm DNA failed to amplify under two out of the three WGA methods (Picoplex and ChromInst), suggesting that under these protocols, the risk of paternal DNA contamination was low, thus making the application of conventional IVF for niPGT-A feasible.

These data suggest that niPGT-A using SBM is a universal approach that can be implemented with minimal changes to standard practice in terms of equipment, consumables, and oocyte/embryo handling.

## 7. Barriers to Implementation of Non-Invasive PGT-A

As outlined above, niPGT-A has several advantages; however, it is also important to consider the limitations of this approach and challenges for implementation.

### 7.1. Concordance: How High Is High Enough?

According to current literature, ploidy concordance can reach as high as 89.6% [88] for SBM-TE pairs, 86.1% [88] for SBM-ICM pairs and 93.8% for SBM-WB pairs [64]. However, scepticism remains regarding whether these figures are sufficient to justify clinical application of niPGT-A.

But assessing the viability of niPGT-A by concordance with conventional PGT-A is not without its limitations. Embryo mosaicism—a phenomenon where two or more genetically different cell lines are present in the embryo [107], confined to either the TE or ICM, or spanning both—can mean that the 5–10 cells analysed in a TE biopsy are not always representative of the embryo as a whole. Should, therefore, the SBM truly be expected to be concordant with the TE in every case? Or, given that the TE is itself concordant with the ICM in 89.6% of embryos—the same percentage as SBM-TE concordance [88], should the same level of concordance between the SBM and the TE be considered justification enough for the application of niPGT-A? Or is concordance a lacklustre metric—should different data, such as clinical outcomes, be the main factor in determining whether the technology is ready to be used as an alternative to conventional PGT-A? These ongoing questions present a significant challenge in the acceptance and implementation of niPGT-A.

### 7.2. Maternal and Exogenous DNA Contamination

One of the main challenges that remains with niPGT-A is the identification of DNA contamination, particularly in female embryos. Due to the low levels of cfDNA found in SBM, even minimal contamination can interfere with the results. There are two main sources of SBM contamination: (i) maternal cells—residual cumulus cells that have not thoroughly been removed during the denudation process—and (ii) exogenous, such as technician/labware contamination and cross-contamination from other embryos. Data from concordance studies show discrepancies in the sex chromosomes between some matched pairs, with the TE biopsy indicating one sex and the SBM showing the opposite—a strong indicator of contamination [61]. Due to the risks associated with the presence of contamination, it is imperative to take all the necessary precautions to minimise its impact. This includes working in a clean area, ideally with materials dedicated for use in the niPGT protocol, wearing personal protective gear, carefully following the embryo culture instructions, such as denudation and serial washes, and including a negative control for each patient.

Research groups are investigating ways in which to account for maternal/external contamination. Current methods require laborious manual assessment of sequencing profiles in the laboratory. Developing an automated approach for contamination detection would be a significant step forward for niPGT.

### 7.3. Result Interpretation and Clinical Guidance

The field of IVF is continuously evolving with an increase in availability and accessibility of genetic testing, meaning that genetic and fertility counselling within the reproductive community has significantly increased over the last two decades. One of the key roles of genetic counsellors is to provide patients with the required resources to make informed decisions about their reproductive treatment. However, in the context of niPGT-A, no guidelines have been developed. Non-invasive PGT-A is a novel technique which is often closely associated with conventional PGT-A; however, sample analysis and results interpretation are very different. The analysis of niPGT-A results must be performed with proprietary algorithms that consider the characteristics of cfDNA. Customised algorithms can be developed to reliably and effectively interpret NGS results by adapting the threshold values for chromosome aneuploidy calling to obtain higher sensitivity and/or specificity when comparing the results with trophectoderm biopsies or whole blastocysts. The niPGT-A results could be influenced by differences in the diagnostic parameters established by different laboratories. This was demonstrated by Huang et al. and Li et al.; both groups reported that ploidy concordance between different embryonic samples varied depending on the cut-off values for mosaicism calling, with optimal results obtained at 60% and 50% thresholds, respectively [59,82]. These data highlight the importance of setting appropriate thresholds that define a chromosome as aneuploid since the criteria applied to TE biopsies and SBM samples can differ.

Regarding interpretation, currently, niPGT-A is not at a stage where it could be considered a diagnostic tool. Instead, it may be applied as an embryo prioritisation method or embryo viability marker that can provide an objective view of an embryo’s chromosomal content—establishing new guidelines for these approaches may be challenging.

These uncertainties raise some ethical questions regarding the adoption of niPGT-A. We have briefly mentioned that niPGT-A could utilise a prioritisation reporting system; however, this may not be universally accepted, with some clinicians wishing to receive detailed chromosomal results. There are several possible methods for reporting niPGT-A, including priority score only or priority score + detailed results for all 24 chromosomes or only for those with viable aneuploidies (13, 18, 21, X, and Y); there may be other reporting variations developed based on regional/legal/clinic-specific demands. The lack of standardisation in reporting begs the question of what happens in cases where only one embryo is analysed and the result is aneuploid—should this embryo receive the highest priority score for transfer since it is the only tested sample? Or perhaps, the aneuploid result should be disclosed to the clinic, even if the chosen method of reporting is based on a priority score alone.

In order to implement niPGT-A reliably and effectively, the differences between conventional and non-invasive PGT-A should be highlighted to clinicians and patients alike. One common debate within the field seeks to determine if there are any circumstances under which clinics should be advised to perform an embryo biopsy following niPGT-A to confirm an aneuploid result—could this be chromosome dependent, medical history dependent, or not recommended at all? Key aspects such as the reporting and management of non-informative results, patients with few to one embryo, and those with advanced maternal age or other medical indications should be carefully discussed to overcome such challenging topics.

Another important detail to consider is the presence and possible implications of embryonic mosaicism. To the best of our knowledge and understanding, there are no specific methods for detecting mosaicism in a non-invasive manner. Nevertheless, as many as 80% of human blastocysts can be considered mosaic [108]. Clinical trials have shown that blastocysts reported as low- or mid-level mosaic (20–50% aneuploid cells) following conventional PGT-A assessment have similar developmental potential as those reported as euploid [107]. However, embryos with high-level mosaicism behave more closely to aneuploid blastocysts, with poorer developmental potential and clinical outcomes [107]. This further emphasises the importance of carefully developing reporting thresholds for aneuploidy calling to ensure that embryos are correctly categorised as euploid or aneuploid. Additionally, clinical implications of embryonic mosaicism, such as live-born offspring with a mosaic chromosomal disorder (e.g., mosaic Down syndrome) [109] or complications associated with confined placental mosaicism (small for gestational age, foetal growth restriction, pregnancy-induced hypertension) [110] should be discussed with patients.

These ethical challenges reinforce the relevance of clinic-based guidelines and transparency from testing providers that cfDNA-based aneuploidy screening is not a direct substitute for conventional PGT-A but instead an objective tool to assist clinicians in determining which embryo should be transferred first.

Indeed, several groups have published high concordance rates, low false positive and false negative rates, and high sensitivity and specificity for niPGT-A, but availability of clinical outcomes, especially focusing on specific patient populations and indication categories, is limited. Using niPGT-A as an objective tool to support morphology-based assessment may be a sensible approach in optimising the chances of successful embryo implantation and pregnancy outcomes.

## 8. Clinical Outcomes

Beyond the initial focus on informativity and concordance, niPGT-A publications have begun to investigate clinical outcomes. Several groups have evaluated clinical outcomes following the transfer of euploid embryos, as determined by SBM analysis only. In a pilot study with 7 single embryo transfers, 5 pregnancies were achieved, resulting in 5 live births [55]. In a second study, 50 transfers of euploid embryos were performed, resulting in a 58% clinical pregnancy rate, with 27 healthy babies born [104].

A retrospective cohort study by Xi et al. has highlighted that SBM analysis can provide useful information to guide embryo transfer. The group published an article with 273 patients with a history of recurrent implantation failure (RIF) and recurrent pregnancy loss (RPL). Clinical outcomes of the study group, in which the patient received euploid embryo transfer according to niPGT-A, performed better vs. the control group, where transfers were carried out based on morphology alone. The clinical pregnancy rate of patients with RIF was 46.9% in the study group in comparison to 28.7% in the control group, whereas the ongoing pregnancy rate of patients with RPL was 40.7% in the study group vs. 25.0% in the control group [68].

Chen et al. and Nakhuda et al. performed 212 and 120 SETs, respectively, based on morphological assessment, showing better pregnancy outcomes for euploid media in comparison to those where the SBM was aneuploid: live birth rate was 46.1% vs. 22.1% and clinical pregnancy rate was 64.0% vs. 44.4%, respectively [111,112].

Most recently, Sun et al. conducted a three-arm study comparing clinical outcomes for transfers based on morphological assessment, niPGT-A, and conventional PGT-A. The group concluded that euploid transfers based on both niPGT-A and conventional PGT-A results increase the cumulative live birth rate in women aged between 35 and 40 years versus morphological assessment [113]. Focusing specifically on comparing clinical outcomes between niPGT-A and conventional PGT-A, Ocali et al. presented data on 409 and 2128 blastocysts that were analysed via each method, respectively [114]. The group reported a similar number of euploid blastocysts between the two groups (niPGT-A: 57.9%; conventional PGT-A: 57.5%), and comparable miscarriage (niPGT-A: 4.3%; conventional PGT-A: 6.2%) and live birth rates (niPGT-A: 65.7%; conventional PGT-A: 61.0%). This study demonstrated that changing current IVF laboratory practice to adopt niPGT-A protocols has no impact on the euploidy rate or clinical outcomes. Similar results were described in good prognosis patients (<38 years) with ongoing pregnancy rates comparable to conventional PGT-A (61.5%) and higher than standalone IVF or ICSI (48.5%) [96]. Combined, these data suggest that niPGT-A can improve clinical outcomes in a similar fashion to conventional PGT-A and better than morphology grading.

Additionally, one group conducted a study using TE biopsy results to guide euploid SETs and retrospectively calculated clinical outcomes in two different scenarios: when euploid TE was concordant with euploid SBM and when euploid TE was discordant with aneuploid SBM. Ongoing implantation rates were three times higher in euploid TE/euploid SBM compared to euploid TE/aneuploid SBM (52.9% vs. 16.7%, respectively), though without reaching statistical significance due to a small sample size. Interestingly, no miscarriages were recorded when TE biopsy and SBM were euploid concordant [61].

Non-invasive aneuploidy testing using SBM, in combination with morphology evaluation, could significantly improve clinical outcomes in ART cycles. A prioritisation-based approach could utilise the top-ranked blastocysts—those with the highest probability of euploidy—for the first transfers, while the remaining embryos could be stored for future transfer, ranked by a combined morphology and aneuploidy score.

Additional research into clinical outcomes would be beneficial, in particular focusing on specific age groups, indications for testing (e.g., recurrent implantation failure, repeated pregnancy loss, male factor), and potential impacts of embryo quality and fertilisation methods.

## 9. Conclusions and Future Work

Overall, we can conclude that analysis of cell-free DNA in the spent blastocyst media could provide objective and helpful information in selecting the best embryo for transfer. Non-invasive PGT-A may provide an effective strategy for embryo prioritisation, with the potential to improve reproductive outcomes when compared to morphological assessment. The precise origin of embryonic cell-free DNA remains unknown, and further study into the mechanisms of cfDNA release is necessary. While informativity and concordance rates have been shown to be high, further clinical outcome data would help drive niPGT-A towards broader implementation. Specific measures in the IVF laboratory are required to maximise the amount of cfDNA present in the final media sample, as well as to reduce the risk of maternal or exogenous contamination; it is important that centres undergo a validation step prior to clinical application to ensure an optimal protocol is followed. These protocol changes do not have a deleterious effect on outcomes. Further consideration is required regarding which patients may benefit the most from niPGT-A testing, along with guidelines for result interpretation, decision making, and embryo prioritisation. Research into niPGT for single-gene disorders (niPGT-M) and structural rearrangements (niPGT-SR) is limited, and initial outcomes have been mixed. Further investigation into using cfDNA for PGT-M/PGT-SR—along with a robust, automatic contamination detection system—would be revolutionary for the field of non-invasive embryo analysis.

## Data Availability

The data presented in this study are available in PubMed at https://pubmed.ncbi.nlm.nih.gov/ accessed on 14 February 2025.
